# 3D Structure Elucidation of Thermostable L2 Lipase from Thermophilic *Bacillus* sp. L2

**DOI:** 10.3390/ijms13079207

**Published:** 2012-07-23

**Authors:** Raja Noor Zaliha Raja Abd. Rahman, Fairolniza Mohd Shariff, Mahiran Basri, Abu Bakar Salleh

**Affiliations:** 1 Faculty of Biotechnology and Biomolecular Sciences, Enzyme and Microbial Technology Research, Universiti Putra Malaysia, UPM Serdang, Selangor 43400, Malaysia; E-Mails: ferrol2506@gmail.com (F.M.S.); abubakar@biotech.upm.edu.my (A.B.S.); 2 Institute of Bioscience, Enzyme and Microbial Technology Research, Universiti Putra Malaysia, UPM Serdang, Selangor 43400, Malaysia; E-Mail: mahiran@science.upm.edu.my; 3 Faculty of Science, Enzyme and Microbial Technology Research, Universiti Putra Malaysia, UPM Serdang, Selangor 43400, Malaysia

**Keywords:** thermostable lipase, L2 lipase, crystallization, counterdiffusion, crystal structure

## Abstract

The crystallization of proteins makes it possible to determine their structure by X-ray crystallography, and is therefore important for the analysis of protein structure-function relationships. L2 lipase was crystallized by using the J-tube counter diffusion method. A crystallization consisting of 20% PEG 6000, 50 mM MES pH 6.5 and 50 mM NaCl was found to be the best condition to produce crystals with good shape and size (0.5 × 0.1 × 0.2 mm). The protein concentration used for the crystallization was 3 mg/mL. L2 lipase crystal has two crystal forms, Shape 1 and Shape 2. Shape 2 L2 lipase crystal was diffracted at 1.5 Å and the crystal belongs to the orthorhombic space group P2_1_2_1_2_1_, with unit-cell parameters a = 72.0, b = 81.8, c = 83.4 Å, α = β = γ = 90°. There is one molecule per asymmetric unit and the solvent content of the crystals is 56.9%, with a Matthew’s coefficient of 2.85 Å Da^−1^. The 3D structure of L2 lipase revealed topological organization of α/β-hydrolase fold consisting of 11 β-strands and 13 α-helices. Ser-113, His-358 and Asp-317 were assigned as catalytic triad residues. One Ca^2+^ and one Zn^2+^ were found in the L2 lipase molecule.

## 1. Introduction

Lipases have became known as one of the leading biocatalysts with established potential for contributing to high potential underexploited lipid technology in bio-industry, and have been used in *in situ* lipid metabolism and *ex situ* industrial applications [1]. Due to the huge successes made in the cloning and expression of enzymes from microorganisms and the increasing demands for these biocatalysts with novel and specific properties such as specificity, stability, pH optimum, and temperature optimum, the number of available lipases has increased since the 1980s [2,3].

Lipases (triacylglycerol acylhydrolase E.C. 3.1.1.3) are a ubiquitous group of enzymes of considerable physiological significance and industrial potential [4]. Lipases catalyze both the hydrolysis of triglycerides to glycerols and free fatty acids over an oil-water interface, and also the hydrolysis and transesterification of other esters as well as the synthesis of esters and exhibiting enantioselective properties [1]. They act at the interface generated by a hydrophobic lipid substrate in a hydrophilic aqueous medium. A characteristic property of lipases is called interfacial activation, meaning a sharp increase in lipase activity observed when the substrate starts to form aggregates such as micelles, thereby presenting an interfacial area to the enzyme [5].

Bacterial lipases are members of the structural superfamily of α/β hydrolases [6] whose enzymic activity results from the catalytic triad Ser-His-Asp, similar to that found in serine proteinases. Generally, the serine residue occurs in the penta-peptide motif of Gly-Xaa-Ser-Xaa-Gly, except in the *Bacillus* lipases where the first glycine is replaced by an alanine. Bacterial lipases have been classified into families with the *Bacillus* lipases falling into subfamily five of family I (I.5). This family consists of lipases from Gram-positive organisms [7].

The thermophilic *Bacillus* sp. L2 previously isolated from a hot spring in Slim River, Perak by Shariff *et al.* (2007) [8], was found to produce a highly thermostable lipase. The *Bacillus* sp. L2 was identified up to genus level based on its morphological, cultural and biochemical characteristics, as well as 16s rDNA gene sequence [9]. L2 was confirmed to be in Group 5 of bacterial classification, a phylogenically and phenotypically coherent group of thermophilic bacilli displaying very high similarity among their 16S rRNA sequences (98.5–99.2%). The thermostable lipase gene was cloned via the PCR cloning method [9]. A high level expression was also successfully achieved by using pQE-30 expression system kit and *Escherichia coli* M15 as expression host. This system has worked efficiently and overexpression of recombinant L2 was successfully carried out with 178-fold activity compared to crude native L2. The optimum pH and temperature were 9.0 and 70 °C, respectively. The enzyme was stable in the pH range of 9.0 to 10.0 for 30 min where the residual activity was retained up to more than 50%. The metal ions Ca^2+^ strongly activated the lipase activity by 100% and 200% when treated with the concentrations 1mM and 5 mM, respectively. Presence of K^+^, Na^+^ and Mn^2+^ also increased the lipase activity by more than 100%. Lipase L2 was strongly inhibited by EDTA (100%), while PMSF, pepstatin A, 2-mercaptoethanol and DTT all showed more than 40% inhibition. Considering the overall properties of different microbial lipases and their evaluations, the thermostable lipase produced by *Bacillus* sp. L2 is an excellent and valuable source of enzyme with regard to its novel properties, such as high thermostablility and stability over a wide range of pH conditions. These properties have proven that this enzyme has a high tolerance level towards harsh environments, thus it is very suitable to be applied in industrial applications.

In this paper, we report the crystallization and the crystal structure of a 43 kDa recombinant thermostable lipase isolated from *Bacillus sp.* L2. The structure possesses the known fold of the a/b hydrolase family, and contains both a calcium ion and a zinc ion.

## 2. Results and Discussion

### 2.1. Purification and Crystallization

**Figure 1:**
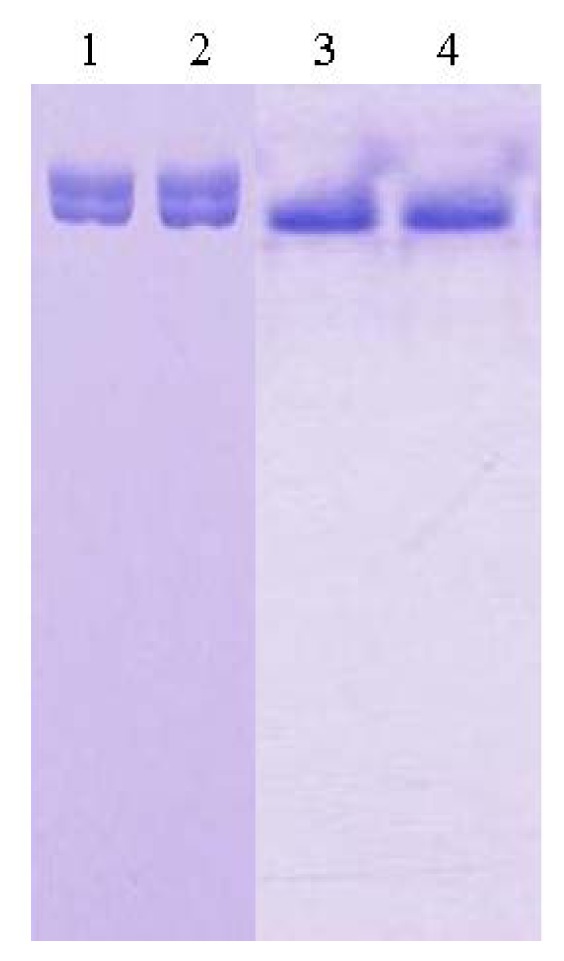
Native-PAGE of purified recombinant L2 lipase. Lanes 1 and 2: L2 lipase purified through Ni-Sepharose affinity column chromatography; Lanes 3 and 4: L2 lipase purified through Ni-Sepharose affinity column chromatography and Super-Q ion exchange column chromatography.

For the purpose of crystallization of L2 lipase, the purification involved two major steps; Ni-Sepharose affinity column chromatography and SuperQ-650S (Tosoh Bioscience, Japan) ion exchange column chromatography (IEX). The desired L2 lipase eluted from this step appeared to migrate as a single band on SDS-PAGE gel but not under native condition. Therefore, the protein was put through an additional ion exchange chromatography step, in order to refine the condition of the purified lipase. Perfect combination of affinity column chromatography Ni-Sepharose and IEX SuperQ-650s had resulted from 49.6 U/mg (crude cell lysate) to 305.3 U/mg (purified L2 lipase) with final recovery of 43.3% obtained from 2L of bacterial culture. The purification was successful and the eluted protein showed a single band on both SDS-PAGE and native-PAGE (Figure 1). Lanes 1 and 2 showed the migrations of L2 lipase purified through Ni-Sepharose affinity column chromatography, while Lanes 3 and 4 showed the migration of L2 lipase purified through Ni-Sepharose affinity column chromatography and SuperQ-650s IEX column chromatography. The highly purified protein was then aliquoted and used for crystallization. For storage purposes, the homogenous protein was kept in a −20 °C freezer to maintain the condition of the protein.

Crystallization of L2 lipase was done using a counter diffusion method in collaboration with JAXA (Japanese Aerospace Exploration Agency) under the JAXA-UPM Protein Crystal Growth (PCG) #1 Flight 1 program. The preliminary crystallization experiment was optimized by using a C-Tube Crystal Tube Kit manufactured by Confocal Science Inc [10]. The optimized crystallization conditions from the C-Tube crystallization experiment were later used in crystallization by using JCB Crystal Tube Kit (Confocal Science Inc). A formulation consisting of 20% PEG 6000, 50 mM MES pH 6.5 and 50 mM NaCl was found to be the best formulation to produce crystals with good shape and size. The protein concentration used for the crystallization was 3 mg/mL. It was found that L2 lipase crystallizes in two forms of crystals; Shape 1 and Shape 2.

**Figure 2:**
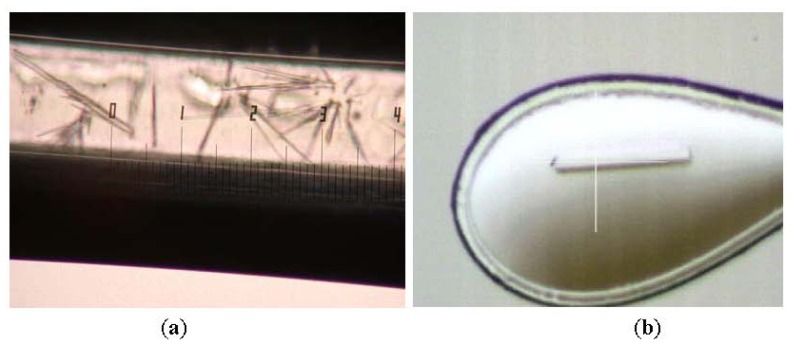
(**a**) Shape 2 L2 lipase crystals in capillary and (**b**) Shape 2 L2 lipase crystal upon diffraction.

In this study, Shape 2 L2 lipase crystals were successfully obtained by using JCB Crystal Tube Kit (Figure 2). The best Shape 2 L2 lipase crystal was diffracted at high resolution (1.5 Å) and belongs to the orthorhombic space group P2_1_2_1_2_1_, with unit-cell parameters a = 72.0, b = 81.8, c = 83.4 Å, α = β = γ = 90°. There is one molecule per asymmetric unit and the solvent content of the crystals is 56.9%, with a Matthew’s coefficient of 2.85 Å^3^ Da^−1^. The data collection and refinement statistics are given separately in Tables 1 and 2, respectively.

### 2.2. Description of Crystal Structure of L2 Lipase

**Figure 3:**
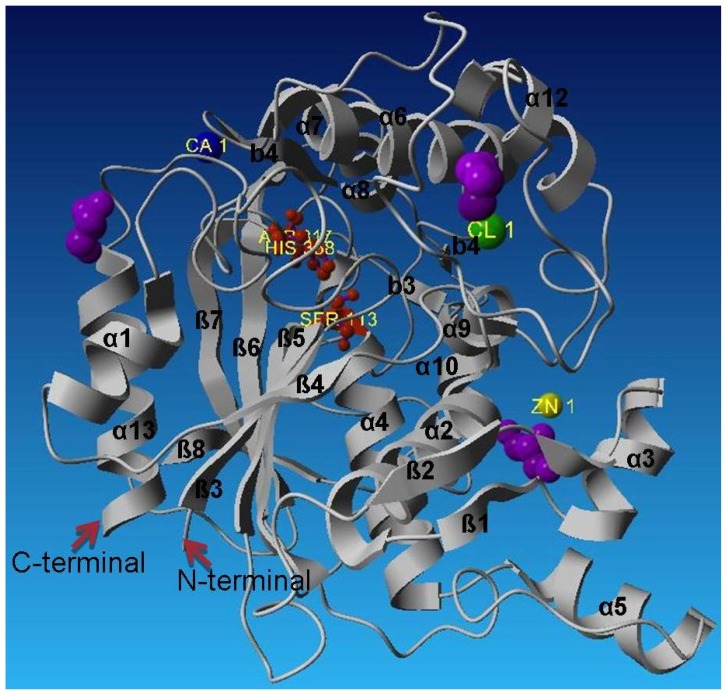
Crystal structure of L2 lipase Shape 2.

L2 lipase Shape 2 molecule has a generally globular shape with approximate dimensions of 20 Å × 25 Å × 33 Å (Figure 2). As seen in other lipase structures, the structure of L2 lipase exhibits similar α/β hydrolase fold. The core structure L2 lipase is made of a seven-stranded parallel sheet that is surrounded by a number of alpha helices; namely alpha helices 1 and 13 on one side, and alpha helices 2, 4, 5, 9,10, 11 and 12 on the opposite site of the structure. In all lipases, a catalytic triad of Ser, His and Asp (or Glu in a few lipases) is present [2]. Based on the structural alignment of L2 lipase with other structurally-known lipases, the Ser113, Asp317 and His358 were assigned as the catalytic triad (Figure 2). The annotated catalytic triad was the same as that derived from the amino acid sequence of L2 lipase. The catalytic serine residue (Ser113) lies in the generously allowed region and situated on the nucleophilic elbow between strand b5 and helix a4 deep within the core structure (Figure 3). This is a typical conformation for the “nucleophilic elbow”, which is positioned in a tightly constrained beta turn type structure between a beta strand and an alpha helix [11].

The catalytic triad Ser 113, His 358 and Asp 317 are shown as red balls and sticks. The Zn^2+^ is the yellow ball, the Ca^2+^ the blue ball and Cl^−^ the green ball. The glycerol molecules are shown in purple. L2 lipase contains the Ala-Xaa-Ser-Xaa-Gly pentapeptide, which is the conserved form in *Bacillus* spp. The pentapeptide of L2 lipase consisted of Ala-His-Ser-Gln-Gly. The active site of L2 lipase is bounded by helix α11 and α12. The lid of the L2 lipase structure is formed by helix α6. The active site of L2 lipase is isolated from solvent by the flexible alpha helical lid helix α6, similar to that found in the structures of P1 lipase (1JI3) [12] and T1 lipase (2dsn) [13]. Helix α6 would most likely be involved in a conformational change that allows the substrate access to the active site [12].

**Figure 4:**
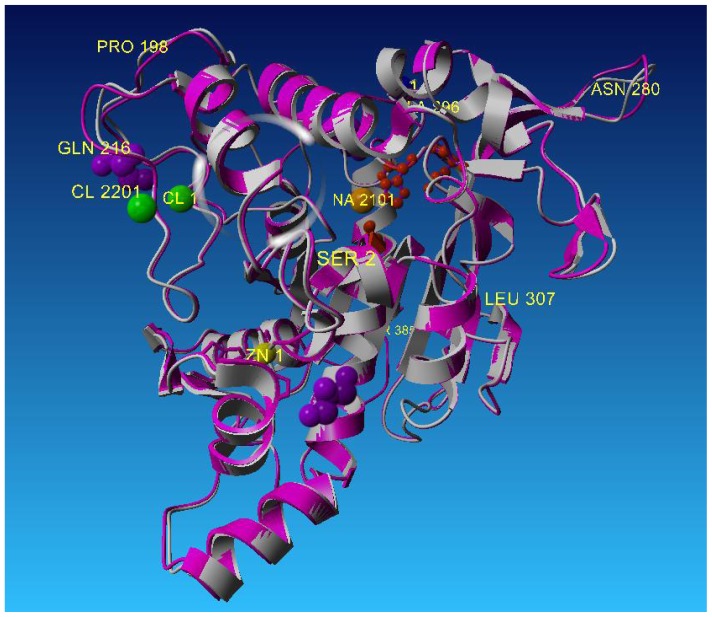
Superposition of L2 lipase Shape 2 Ground with 2DSN (T1 Lipase), with value of rmsd = 0.76 Å.

T1 lipase has high homology with L2 lipase with only one amino acid difference at the C-terminal of the protein sequence as shown in Table 3. Thus, T1 lipase was used as a search template in this study. Based on their crystal structures, only minor differences could be seen when superimposed. The differences were found at a number of turn positions of the structures which were composed of the same amino acids but with different rotation or translation side chains. The crystal structure of L2 lipase showed an RMS deviation of 0.76Å when superimposed by Cα traces with crystal structure of T1 lipase (2dsn) (Figure 4).

### 2.3. Metal-Binding Sites

**Figure 5:**
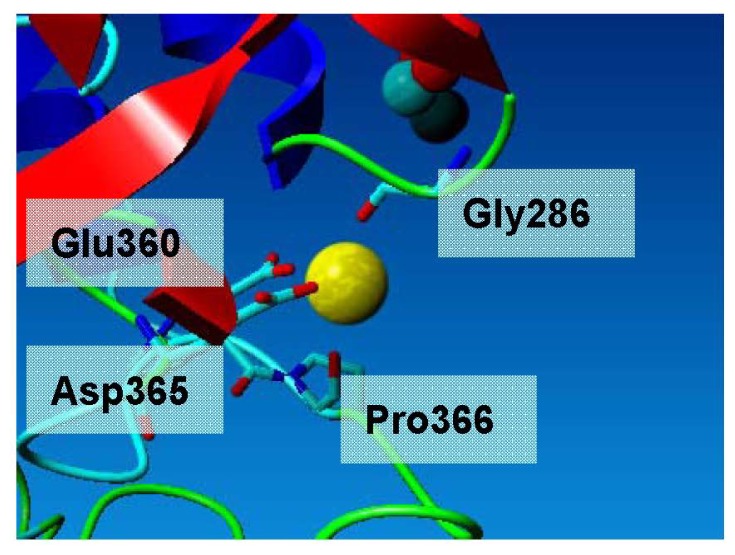
Calcium ion binding site of L2 lipase. The Ca^2+^ is coordinated in an octahedral environment via Gly286 O, Glu360 O Asp365 O, Pro366 O and two water molecules. All calcium–ligand distances are approximately 2.4 Å, which is in accordance with reported values.

L2 lipase crystal structure Shape 2 revealed the presence of Ca^2+^ and Zn^2+^ attached with its molecule. L2 lipase has a structural motif of Ca-binding EF-hand. The shape was perceived as a hand with extended fingers that cups the Ca^2+^ [14]. A typical Ca-binding EF-hand motif is coordinated by a backbone oxygen, a water atom, two carbonyl oxygen atoms of Asp residues, the amide oxygen of Gln residue and the forked carboxyl group of glutamate residue. The interactions are a quite complex mixture of ionic and polar dipole interactions, and although there are seven atoms around calcium, distances indicate that the bifurcated contact to the Glu residues has a large ionic component, with the sixth corner of the octahedron presenting the point of highest negative partial charge density. In the L2 lipase structure, the Ca^2+^ is coordinated in an octahedral environment via Gly286 O, Glu360 O Asp365 O, Pro366 O and two water molecules (Figure 5). All calcium–ligand distances are approximately 2.4 Å, which is in accordance with reported values.

**Figure 6:**
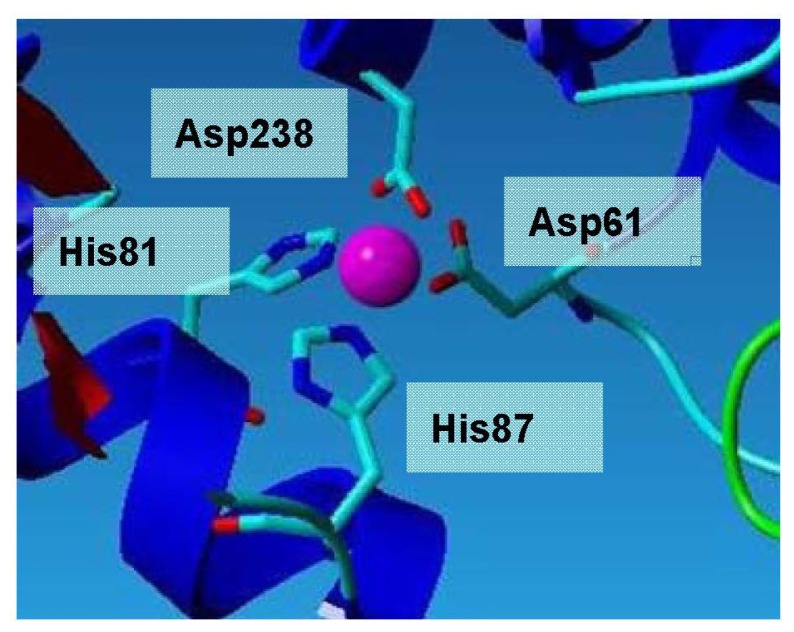
Zinc ion binding site of L2 lipase. All zinc-ligand distances are between 2.0 Å and 2.2 Å, which are as expected for zinc.

The Zn^2+^ is coordinated in a tetrahedral environment by two histidine N12 atoms (His81 and His87) located in helix α3, and two aspartate O atoms (Asp61 and Asp238) (Figure 6). All zinc-ligand distances are between 2.0 Å and 2.2 Å, which are as expected for zinc. The Zn^2+^ is approximately 19 Å away from the catalytic serine residue, and is thus unlikely to participate in catalysis. Instead, it could be suggested that it plays a role in conferring enhanced stability [12]. The zinc-binding motif consists of two histidines and two aspartic acid residues.

## 3. Experimental Section

### 3.1. Purification of L2 Lipase

The gene-encoding thermostable lipase L2 was isolated from *Bacillus* sp. L2 that had been collected from a hot spring in Slim River, Malaysia. The enzyme was over-expressed in *E. coli*, and the single-step Ni-Sepharose affinity chromatography work were described previously [9]. For crystallization purposes, the purified L2 lipase was put through another additional chromatography step—a super-Q Tosoh ion exchange chromatography step—in order to obtain refined, homogenous protein with the same charge distribution on its surface.

### 3.2. Crystallization and Data Collection

L2 lipase was crystallized by using a counter diffusion method using J-tube and JCB tube [10]. It was found that L2 lipase crystal has two forms of crystals; Shape 1 and Shape 2. The high resolution structure of Shape 2 (1.5 Å resolution) L2 lipase crystal belong to the orthorhombic space group P2_1_2_1_2_1_, with unit-cell parameters a = 72.0, b = 81.8, c = 83.4 Å, α = β = γ = 90°. There is one molecule per asymmetric unit and the solvent content of the crystals is 56.9%, with a Matthew’s coefficient of 2.85 Å^3^ Da^−1^.

Thermostable L2 lipase crystals were diffracted at the SPring-8 BL41XU beamline, Hyogo Prefecture, Japan. The synchrotron is operated at 8 GeV with a wavelength of 0.98 Å. The detector used was ADSC Quantum-315 CCD Detector. The cryo-protected crystals were flash-frozen in a N2 stream at 277 K prior to diffraction. The oscillation range used was 1°. The data collections of the diffraction were indexed, integrated and reduced by using DENZO and SCALEPACK from the HKL2000 package program.

### 3.3. Structure Solution and Crystallographic Refinement

The structure solution of L2 lipase was done by using the CCP4 program package [14] and the Coot program [15]. The phase problem was solved by using a molecular replacement method by using the Phaser program. To do the molecular replacement, the crystal structure of T1 lipase (2DSN) which has high homology (99% sequence identity) with L2 lipase was used as a search template. These files were then used in the refinement process. Refinement was done using Refmac program [16] in the CCP4 program. The electron density map was viewed using the program Coot. The solvent molecules were inserted using this program with automatic determination of statistically significant density levels for inclusion of new water molecules.

### 3.3. Protein Data Bank ID Code

The atomic coordinates and structure factors have been deposited with the RCSB Protein Data Bank as entry 4FDM

## 4. Conclusions

In this study, we reported the crystallization of thermostable L2 lipase by utilizing the counter diffusion method, the diffraction of L2 lipase crystal and the 3-D crystal structure of L2 lipase crystal. Shape 2 crystal of L2 lipase was successfully diffracted at 1.5 Ǻ. Complete data collection was collected and comparison with reports from available literature showed that this is the only thermostable lipase solved at high resolution that belongs to the orthorhombic space group P2_1_2_1_2_1_, as the other thermostable lipases such as P1 lipase, T1 lipase and L1 lipase all belong to the monoclinic C2 space group [10,12,13,18]. Change in the space group of L2 lipase happens, despite a difference of only 1–5 amino acids with the other thermostable lipases. The contact regions between the neighboring molecules and these different amino acids may affect the conformations of the lipases as a result of crystal packing. A different space group suggested that the packing of L2 lipase is different with other thermostable lipases mentioned in this manuscript. Despite the fact that the core structure is almost similar, flexible and dynamic regions can be fixed in a specific conformation of the particular enzyme because of crystal packing interactions and altered conformations of flexible regions may be induced. In addition, it is the only thermostable lipase crystal that was successfully crystallized by the counter diffusion crystallization method [10]. The 3-D crystal structure of L2 lipase showed that L2 lipase exhibits a similar α/β hydrolase fold as seen in other lipase structures. The Ser113, Asp317 and His358 were assigned as the catalytic triad. The structure has two metal ions; Ca^2+^ and Zn^2+^.

## Figures and Tables

**Table 1:** Statistics of data collection for L2 lipase Shape 2 Ground crystal diffracting to 1.5 Å. Values in parentheses are for the highest resolution shell (1.50–1.49).

Crystal Data	
Space group	P2_1_2_1_2_1_
Resolution range Unit cell parameter (Å)	40.00–1.49
a	71.98
b	81.78
c	83.37
Mosaicity range (°)	0.14–0.29

**Data Processing**	

Temperature (K)	100
Wavelength (Å)	0.80
Resolution (Å)	40.00–1.49
Total number of reflections	827852
Oscillation angle per frame (°)	0.3
Unique data	81336
Redundancy (%)	4.05 (4.23)
Data completeness (%)	99.50 (97.90)
Mean I/Sigma	20.50 (2.87)
Molecules per ASU	1
Matthews coefficient, Å^3^ Da^−1^	2.85
Solvent content (%)	56.89
Total linear R_merge_	0.098

**Table 2:** Refinement statistics for L2 lipase.

Refinement Statistics	L2 Shape 2
Molecular per ASU	1
Resolution Range (Å)	58.4–1.5
Protein non-hydrogen atoms	3037
Ligand non-hydrogen atoms	-
Glycerol	3
Water molecules	497
Metal ions	2
Rfactor (%)	0.167
Rfree (%)	0.172
R.m.s.d. bond length (Å)	0.009
R.m.s.d. bond angle (°)	1.20
Average B-factor	15.01

**Table 3:**
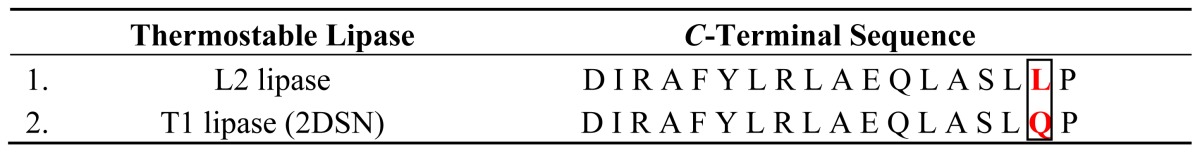
Amino acid differences between L2 lipase with T1 lipase.

## References

[1] Triechel H., de Oliveira D., Mazutti A.M., Luccio M.D., Oliveira J.V. (2010). A review on microbial lipases production. Food Bioproc. Technol.

[2] Bornscheuer U.T., Bessler C., Srinivas R., Krishna S.H. (2002). Optimizing lipases and related enzymes for efficient application. Trends Biotechnol.

[3] Menoncin S., Domingues N.M., Freire D.M.G., Toniazzo G., Cansian R.L., Oliveira J.V. (2008). Study of the extraction, concentration, and partial characterization of lipases obtained from *Penicillium verrucosum* using solid-state fermentation of soybean bran. Food Bioproc. Technol.

[4] Sharma R., Soni S., Vohra R., Gupta L., Gupta J. (2002). Purification and characterization of a thermostable alkaline lipase from a new thermophilic *Bacillus* sp. RSJ-1. Proc. Biochem.

[5] Jaeger K.E., Ransac S., Dijkstra B.W., Clson C., Heuvel M.V., Misset O. (1994). Bacterial lipases. FEMS Microbiol. Rev.

[6] Ollis D.L., Cheah E., Cygler M., Dijkstra B., Frolow F., Ken S.M. (1992). The α/β hydrolase fold. Protein Eng.

[7] Arpigny J.L., Jaeger K.-E. (1999). Bacterial lipolytic enzymes: Classification and properties. Biochem. J.

[8] Shariff F.M., Chor A.L.T., Mukred A.D., Salleh A.B., Basri M., Rahman R.N.Z.R.A. (2007). Production of L2 lipase by *Bacillus* sp. strain L2: nutritional and physical factors. J. Basic Microbiol.

[9] Shariff F.M., Rahman R.N.Z.R.A., Basri M., Salleh A.B. (2011). A newly isolated thermostable lipase from *Bacillus* sp. Int. J. Mol. Sci.

[10] Shariff F.M., Rahman R.N.Z.R.A., Ali M.S.M., Chor A.L.T., Basri M., Salleh A.B. (2010). Crystallization and preliminary X-ray crystallographic analysis of a highly thermostable L2 lipase from a newly isolated *Bacillus* sp. L2. Acta Crystallogr. F.

[11] Dodson G., Wlodawer A. (1998). Catalytic triads and their relatives. Trends Biochem. Sci.

[12] Joel D.A., Tyndall J.D.A., Sinchaikul S., Fothergill-Gilmorel L.A., Tayor P., Walkinshaw M.D. (2002). Crystal structure of a thermostable lipase from *Bacillus stearothermophilus* P1. J. Mol. Biol.

[13] Matsumura H., Yamamoto T., Chor A.L.T., Mori T., Salleh A.B., Basri M., Inoue T., Kai Y., Rahman R.N.Z.R.A. (2007). Novel cation-pi interaction revealed by crystal structure of thermoalkalophilic lipase. Proteins.

[14] Lewit-Bentley A., Réty S. (2000). EF-hand calcium-binding proteins. Curr. Opin. Struct.

[15] Collaborative Computational Project, Number 4 (1994). The CCP4 suite: Programs for protein crystallography. Acta Crystallogr.

[16] Emsley P., Lohkamp B., Scott W.G., Cowtan K. (2010). Features and development of coot. Acta Crystallogr. Biol. Crystallogr.

[17] Murshudov A.A., Vagin A.A., Dodson E.J. (1997). Refinement of macromolecular structures by the maximum-likelihood method. Acta Crystallogr. D.

[18] Jeong S.-T., Kim H.-K., Kim S.-J., Pan J.-G., Oh T.K., Ryu S.E. (2001). Crystallization and preliminary X-ray crystallographic analysis of a thermoalkalophilic lipase from *Bacillus stearothermophilus* L1. Acta Crystallogr. D.

